# A Loss to Mental Science

**Published:** 1889-01

**Authors:** 


### A LOSS TO MENTAL SCIENCE.

A Chicago woman has gone crazy under the mind-cure treatment of two local "metaphysicians." Brethren, hers is evidently the very kind of a mind that that kind of a mind-cure fits. It is a great pity that she went crazy. She seems to have been just enough of an imbecile to have completed the regular "metaphysical" university course in three weeks, and then, great Webster! what a teacher she would have made. Well, well; we suppose there must always be some asses in the world. The money invested in asylums for the feeble-minded would be a great loss if all the fools should suddenly have a spasm of common sense and die; two things which no fool was ever yet known to do. The Emperor William dies, Garfield dies, Hendricks dies, Conkling dies, Grant and Lincoln die; but the people who could die a great deal better and more acceptably than they live; the people who live merely for the the purpose of taking up room, the people whose places in the world could be supplied most easily by a set of clothes hooks, and an automatic digestive apparatus, they live on and on and on and on and on.—*Burdette.*

